# When Bodybuilding Goes Wrong—Bilateral Renal Artery Thrombosis in a Long-Term Misuser of Anabolic Steroids Treated with AngioJet Rheolytic Thrombectomy

**DOI:** 10.3390/ijerph19042122

**Published:** 2022-02-14

**Authors:** Artur Lemiński, Markiian Kubis, Krystian Kaczmarek, Adam Gołąb, Arkadiusz Kazimierczak, Katarzyna Kotfis, Marcin Słojewski

**Affiliations:** 1 Department of Urology and Urological Oncology, Pomeranian Medical University, al. Powstańców Wlkp. 72, 70-111 Szczecin, Poland; mv.kubis@gmail.com (M.K.); k.kaczmarek.md@gmail.com (K.K.); adamgol@cyberia.pl (A.G.); mslojewski@gmail.com (M.S.); 2 Department of Vascular Surgery and Angiology, Pomeranian Medical University, al. Powstańców Wlkp. 72, 70-111 Szczecin, Poland; biker2000@icloud.com; 3 Department of Anesthesiology, Intensive Therapy and Acute Intoxications, Pomeranian Medical University, al. Powstańców Wlkp. 72, 70-111 Szczecin, Poland

**Keywords:** doping in sports, public health, testosterone, stanozolol, thrombosis, renal infarction, acute kidney injury, thrombolytic therapy, nephrectomy

## Abstract

Bilateral renal infarction is an extremely rare condition with only few cases reported in the literature. We present a case of bilateral renal infarction affecting an otherwise healthy 34 year old bodybuilder chronically misusing testosterone and stanozolol. The patient presented with severe flank pain mimicking renal colic and biochemical features of acute kidney injury. Diagnostic workup revealed thrombosis affecting both renal arteries. Subsequently, the patient underwent a percutaneous rheolytic thrombectomy with AngioJet catheter, along with catheter-directed thrombolysis. Right-sided retroperitoneal hematoma developed as an early complication, mandating surgical exploration and nephrectomy due to kidney rupture and the unstable condition of the patient. Intensive care and continuous renal replacement therapy were instigated until a gradual improvement of the patient status and a return of kidney function was achieved. No abnormalities were found in the cardiological and hematological evaluation. We believe this is a first report of bilateral renal infarction associated with anabolic steroid misuse in an otherwise healthy individual, and a first report of AngioJet thrombectomy in bilateral thrombosis of renal arteries. It stresses the importance of a thorough diagnostic workup of colic patients and emphasizes the need for sports medicine to reach out to amateur athletes with education on the harms of doping.

## 1. Introduction

Renal infarction (RI) is a rare condition that occurs when kidney vascularization becomes severely compromised, often leading to a loss of the renal unit. Causative factors for RI include atrial fibrillation, renal artery stenosis or dissection, inherited hypercoagulative states, amyloidosis and trauma [1,2,3,4,5]. There are scarce reports on bilateral RI caused by fibromuscular dysplasia, hereditary thrombophilia, cocaine abuse and severe COVID-19 [2,6,7,8]. However, to the best of our knowledge, no case of bilateral RI linked to bodybuilding and long-term misuse of anabolic androgenic steroids (AAS) has been published to date.

## 2. Case Presentation

**Figure 1:**
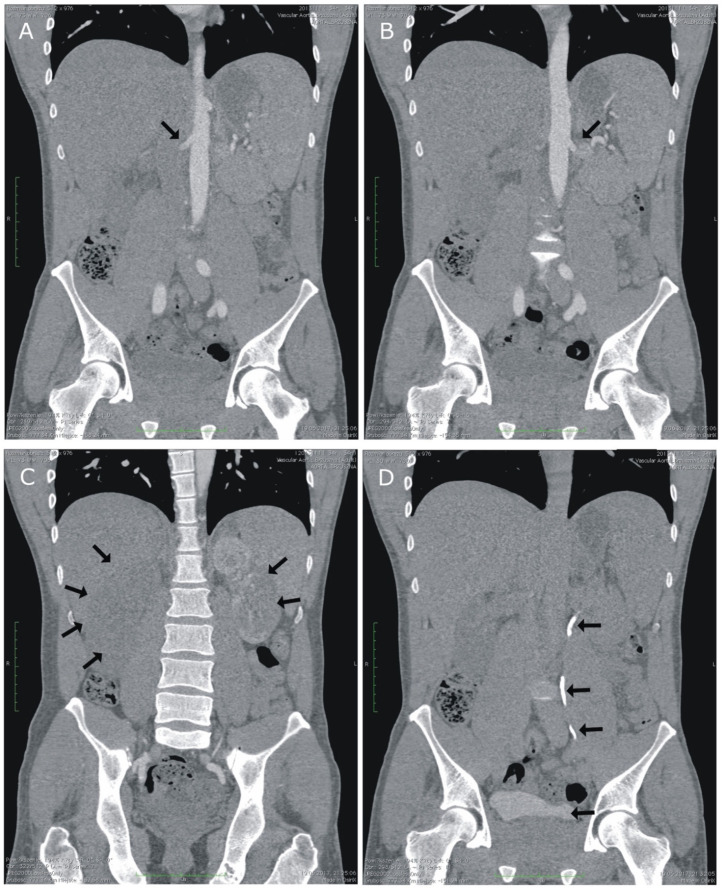
(**A**,**B**) Angio-computed tomography scans showing areas of thrombosis in the renal arteries. (**C**) Infarction (not enhancing) areas within the right and left kidneys. (**D**) Preserved excretion of contrast urine by intact portions of the left kidney parenchyma.

We present a case of an otherwise healthy 34 year old male bodybuilder who presented to the emergency department due to severe right flank pain not responding to analgesics, lasting for 2 h. The medical history revealed use of AAS (testosterone and stanozolol) for several months as a part of his bodybuilding program but was otherwise unremarkable with no familial history of thromboembolic events. The physical examination revealed right flank percussion tenderness, atrophic testicles, extensive muscle mass and reduced adipose tissue. The routine laboratory evaluation showed a white blood cells count of 12.23 × 10^9^/L, thrombocytosis of 478 × 10^9^ /L, C-reactive protein of 19.3 mg/L, D-Dimer of 988.1 ng/mL, creatinine of 1.82 mg/dL, proteinuria of 1200 mg/L and up to 10 erythrocytes per high power field in the urinary sediment. The routine clotting parameters, i.e., an activated partial thromboplastin time (APTT) of 27.6 s, prothrombin time (PT) of 11.8 s and international normalized ratio (INR) of 1.07 were within normal ranges. The kidney ultrasound showed no signs of nephrolithiasis or hydronephrosis, but the Doppler examination revealed blood flow to be restricted to the proximal part of the right renal artery (RA). An abdominal angio-Computed Tomography (angio-CT) scan was performed, revealing bilateral thrombosis of RAs, with only a scant perfusion preserved in the upper pole of the left kidney (Figure 1A–D).

The AngioJet (Boston Scientific, Marlborough, MA, USA) rheolytic thrombectomy of both RAs and intra-arterial thrombolysis with 20 mg of alteplase were performed, restoring their patency, yet the parenchyma of the right kidney was only partly enhancing, and no venous return was seen. Additionally, an iatrogenic injury to the right lower pole segmental artery occurred. Despite attempts at endovascular management, a progressive decline in the hemoglobin level and deterioration of the patient’s status were observed postoperatively. The emergency ultrasound confirmed the presence of right-sided retroperitoneal hematoma requiring urgent surgical revision. Intraoperatively, the rupture of kidney parenchyma and extensive macroscopic features of necrosis were found within the right kidney, which, given the unstable patient condition, mandated an immediate nephrectomy. The patient required admission to the intensive care unit and continuous renal replacement therapy due to clinical manifestations of acute kidney injury. He received a transfusion of five units of packed red blood cells. Subsequently, a progressive improvement of the clinical status and increase in the urine output were observed in the following days, along with a gradual lowering of serum creatinine (Table 1). 

The patient underwent a cardiological evaluation with resting echocardiography which revealed a left ventricle hypertrophy but no thrombotic material within heart cavities. The hematological evaluation has not revealed any inherited clotting disorders leading to hypercoagulability. The patient was discharged home prematurely, at his own request, with decreasing creatinine on low molecular weight heparin. After four years of follow-up, the patient is well, with no significant deterioration of the glomerular filtration rate (GFR) observed over time. He has not suffered any further thromboembolic events and is not medicated with anticoagulant. He undergoes a systematic control with his general practitioner and nephrologist. According to the information provided by the patient, a thrombophilia screen performed outside of our center tested negative and included antithrombin III, protein C, protein S, lupus anticoagulant, factor V Leiden, prothrombin gene mutation, anti-β-2-glycoprotein-1 antibodies and anti-cardiolipin antibodies.

## 3. Discussion

Amateur sport offers unrestrained access to a plethora of dietary supplements and medications, including AAS. Their chronic abuse is widespread among young bodybuilders, with consequences reaching far beyond muscle growth [9]. The spectrum of health sequelae of long-term AAS abuse predominantly affects men of a productive age and therefore constitutes an increasing public health concern [10]. These include but are not limited to cardiovascular toxicity, infertility, psychiatric disorders, withdrawal-associated hypogonadism and induced thrombophilia. Animal studies revealed that prolonged AAS administration intensifies platelet aggregation through increased production of thromboxane A2 and reduced biosynthesis of prostacyclin [11]. Furthermore, chronic AAS exposure induces polycythemia, retention of fluids and vasoconstriction, contributing to the development of hypertension. On top of that, AAS hasten the progress of atherosclerosis due to alterations in the lipid metabolism, which further contributes to an increased risk of thrombosis [12]. Given the detrimental effects of chronic AAS intake on the myocardium leading to left ventricle hypertrophy and diastolic impairment, the long-term misuse of these agents makes affected athletes and bodybuilders predisposed to cardiovascular complications [13]. Specifically, stanozolol combinations have been reported to increase the risk of cardiovascular and thrombotic events, being associated with cases of myocardial infarction, ischemic stroke, severe peripheral arterial thrombosis as well as sudden cardiac death in young bodybuilders [14,15,16,17].

Patients with RI may present with a nonspecific constellation of signs and symptoms, usually resembling renal colic [18]. Acute flank pain should trigger a thorough diagnostic workup with ultrasound, including a Doppler study as a main screening modality. Angio-CT and angio-MRI have largely replaced conventional angiography as noninvasive imaging options for a rapid assessment of the patency of RAs and perfusion of kidney parenchyma [19]. Unilateral RIs of limited extent without associated AKI are usually amenable to conservative management with anticoagulants [3,20]. Treatment of extensive or bilateral RI should include percutaneous transluminal angioplasty, AngioJet thrombectomy or catheter-directed thrombolysis, which allow for a successful reperfusion in the majority of cases. Rheolytic thrombectomy with AngioJet catheters has been successfully employed for a broad spectrum of thrombotic and embolic complications in both elective and emergency settings [21,22]. There have been several reports of successful applications of AngioJet in cases of renal infarction due to renal artery thrombosis or emboli; nonetheless, no case of kidney rupture after reperfusion has been described [23,24,25]. It remains undetermined whether the rupture of the right kidney after the restoration of the patency of RI occurred as a result of concomitant renal vein thrombosis, similarly to several reports of renal allograft ruptures, or due to intraoperative injury at the time of the thrombectomy [26,27]. Notwithstanding this, the remaining left kidney was reperfused successfully, and no significant deterioration of the creatinine level or GFR was observed in the subsequent four years of follow-up.

## 4. Limitations

This case report has some limitations that need to be acknowledged. The patient was discharged home prematurely at his own request. Consequently, the hematological evaluation for clotting disorders could not be accomplished during his hospital stay at our center with the panel usually including antithrombin III, protein C, protein S, lupus anticoagulant and factor V Leiden. Therefore, the thrombophilia screening was performed in his place of residence abroad. Given limited access to his medical files, we only managed to gather the information provided by a telephone follow-up from the patient (as per information from the GP), according to which no inherited clotting disorders leading to a hypercoagulable state were diagnosed and the performed thrombophilia screen included antithrombin III, protein C, protein S, lupus anticoagulant, factor V Leiden, prothrombin gene mutation, anti-β-2-glycoprotein-1 antibodies and anti-cardiolipin antibodies. The patient required no anticoagulant treatment. Moreover, the exact dosage and timespan of AAS administration remains unknown, as these were given non-professionally at the gym and no records were made. The patient reported the AAS use as being inconsistent and variable, but nonetheless lasting several months. Notwithstanding this, it seems plausible that recreational AAS abuse was pathogenetically linked to this rare thrombotic episode.

## 5. Conclusions

Great care is needed in order not to overlook RI in a colic patient, particularly when no hydronephrosis is found and risk factors for thrombosis are present. Furthermore, widespread AAS abuse constitutes a growing public health problem and emphasizes an unmet need for education on the harms of doping among amateur athletes and bodybuilders. The rheolytic thrombectomy with an AngioJet catheter remains a viable treatment option for extensive or bilateral renal infarction.

## Figures and Tables

**Table 1:** Serum creatinine, estimated glomerular filtration rate and 24 h urine output during hospital stay.

Day of Stay	1	2	3	4	5	6	7
Creatinine [mg/dL]	1.82	2.66	3.82	4.34	4.98	4.43	3.89
eGFR [mL/min/1.73 m^2^]	47	30	19	17	14	16	19
Urine output [mL]	n.d.	2100	450	780	4500	8400	2900

eGFR: estimated glomerular filtration rate.

## Data Availability

Data will be available from the corresponding author upon reasonable request.
